# Urinary Cadmium Threshold to Prevent Kidney Disease Development

**DOI:** 10.3390/toxics6020026

**Published:** 2018-05-01

**Authors:** Soisungwan Satarug, Werawan Ruangyuttikarn, Muneko Nishijo, Patricia Ruiz

**Affiliations:** 1 National Research Centre for Environmental Toxicology, the University of Queensland, Brisbane 4108, Australia; sj.satarug@yahoo.com.au; 2 UQ Diamantina Institute and Centre for Health Services Research, Centre for Kidney Disease Research and Translational Research Institute, Woolloongabba, Brisbane 4102, Australia; 3 Division of Toxicology, Department of Forensic Medicine, Chiang Mai University, Chiang Mai 50200, Thailand; ruangyuttikarn@gmail.com; 4 Department of Public Health, Kanazawa Medical University, Uchinada, Ishikawa 920-0293, Japan; ni-koei@kanazawa-med.ac.jp; 5 Computational Toxicology and Methods Development Laboratory, Division of Toxicology and Human Health Sciences, Agency for Toxic Substances and Disease Registry, Centers for Disease Control and Prevention, Atlanta, GA 30333, USA

**Keywords:** β2-microglobulin, cadmium, chronic kidney disease, clinical kidney function measure, estimated glomerular filtration rate, *N*-acetyl-β-d-glucosaminidase, population health, tubular dysfunction, toxicity threshold limit, urine protein

## Abstract

The frequently observed association between kidney toxicity and long-term cadmium (Cd) exposure has long been dismissed and deemed not to be of clinical relevance. However, Cd exposure has now been associated with increased risk of developing chronic kidney disease (CKD). We investigated the link that may exist between kidney Cd toxicity markers and clinical kidney function measure such as estimated glomerular filtration rates (eGFR). We analyzed data from 193 men to 202 women, aged 16−87 years [mean age 48.8 years], who lived in a low- and high-Cd exposure areas in Thailand. The mean (range) urinary Cd level was 5.93 (0.05–57) μg/g creatinine. The mean (range) for estimated GFR was 86.9 (19.6−137.8) mL/min/1.73 m^2^. Kidney pathology reflected by urinary β2-microglobulin (β2-MG) levels ≥ 300 μg/g creatinine showed an association with 5.32-fold increase in prevalence odds of CKD (*p* = 0.001), while urinary Cd levels showed an association with a 2.98-fold greater odds of CKD prevalence (*p* = 0.037). In non-smoking women, Cd in the highest urinary Cd quartile was associated with 18.3 mL/min/1.73 m^2^ lower eGFR value, compared to the lowest quartile (*p* < 0.001). Evidence for Cd-induced kidney pathology could thus be linked to GFR reduction, and CKD development in Cd-exposed people. These findings may help prioritize efforts to reassess Cd exposure and its impact on population health, given the rising prevalence of CKD globally.

## 1. Introduction

Exposure to the heavy metal cadmium (Cd) is inevitable for most people as this metal is present in foodstuffs, cigarette smoke and polluted air [1,2,3,4]. By total diet studies, staple foods such as rice, potatoes, and wheat constitute 40–60% of total dietary Cd intake in the average consumer in various populations [4]. In addition, offal, spinach, shellfish, crustacean and mollusks constitute dietary Cd sources [4]. Cd oxide (CdO) in cigarette smoke and polluted air has relatively high bioavailability. Consequently, most smokers show elevated Cd levels in their blood, urine, and tissues [1,2,3,4]. To-date, non-occupational Cd exposure has been associated with numerous chronic diseases of continuously rising prevalence, notably type-2 diabetes [2,3,4]. However, the most frequently reported Cd toxicity in non-occupationally exposed populations is related to kidneys, notably the injury to the proximal tubular epithelial cells that reabsorb and concentrate Cd from the glomerular filtrate [1,2,3,4]. Renal tubular cells are highly susceptible to Cd-induced apoptosis because of high abundance of mitochondria and substantial reliance on autophagy to maintain homeostasis [5,6,7]. One of the consequential results of the injury and death of renal tubular epithelial cells by Cd is a reduction in tubular reabsorption capacity in Cd-exposed people, leading to loss of nutrients through urine, notably glucose, amino acids, calcium, and zinc [8,9,10,11].

Urinary levels of *N*-acetyl-β-d-glucosaminidase (NAG) enzyme and the low molecular weight protein β2-microglobulin (β2-MG) are often used to reflect Cd-induced kidney tubular pathologies [12,13,14,15], while urinary Cd excretion is used as an indicator of cumulative long-term exposure or body burden [16,17,18]. For example, elevated urinary β2-MG levels (≥283 μg/day) were reported for subjects who excreted 3.05 μg of Cd per day [8]. In Japanese studies, urinary Cd levels 1.6–4.6 μg/g creatinine were associated with urinary β2-MG levels ≥ 1000 μg/g creatinine, an indicative of severe and irreversible tubular dysfunction [19,20]. However, these signs of Cd-related tubular toxicity have not been considered to be clinically relevant.

Challenging the notion on a lack of clinical and health risk implications are data from the representative of Korean population, and from two cycles of the U.S. National Health and Nutrition Examination Surveys (NHANES) showing that Cd exposure may increase the risk of developing chronic kidney disease (CKD) [21,22,23,24]. Dietary Cd intake has also been associated with CKD development in Chinese population [25]. However, none of these studies has assessed glomerular filtration rate (GFR) concurrently with kidney tubular pathology markers, notably urine NAG and β2-MG. We hypothesize that GFR falls as the result of Cd destroys tubular cells after reabsorption from filtrate. Hence, the present study investigated the potential link between Cd tubular toxicity and CKD in Cd-exposed Thai subjects. We sought to evaluate an independent association between GFR reduction and evidence of tubular pathologies in relation to urinary Cd levels, age, gender, body mass index (BMI), and smoking.

## 2. Methods

### 2.1. Study Subjects

To represent chronic environmental exposure situations, we assembled a group of 395 subjects from a low-Cd exposure area in Bangkok [26], and high-Cd exposure area in rural rice farming villages in Mae Sot District, known to be an area with Cd contamination [12,27]. Subjects in neither low-Cd nor high-Cd exposure group were occupationally exposed to metals. The Institutional Ethical Committee, Chulalongkorn University Hospital, approved the Bangkok study protocol, while the Mae Sot Hospital Ethical Committee approved the Mae Sot study protocol. All participants provided informed consent prior to participation. For a low-Cd exposure group, inclusion criteria were apparently healthy. Exclusion criteria were pregnancy, breast-feeding, history of metal work, a hospital record or diagnosis by physician of CKD, heart disease, diabetes, anemia, or hyperlipidemia. For a high-Cd exposure group, subjects were randomly selected from 13 villages with cadmium pollution in Mae Sot District, Tak Province. There were cases of diagnosed CKD, hypertension, and osteoporosis as shown in Table 1. Smoking, regular use of medications, education, occupation, family health history and anthropometric data were obtained from questionnaires. Excluding those with incomplete data, 395 subjects (180 from the Bangkok group, and 215 from the Mae Sot group) form study subjects in the present study.

### 2.2. Ascertainment of Long-Term Cadmium Exposure Levels

Assessment of long-term Cd exposure or body burden was based on creatinine-adjusted urinary Cd concentrations. Urinary Cd is a suitable exposure marker to assess kidney effects since the majority of Cd in urine is ultrafilterable, but not reabsorbed by kidney tubules [2]. The plasma Cd concentration reflects Cd influx into blood circulation from external sources (diet and air) and internal reservoirs (liver). Accordingly, urinary Cd excretion rate is proportional to plasma Cd concentrations, glomerular filtration rates and tubular sequestration rates [2]. For the Bangkok group [26], the urinary Cd concentrations were determined with the inductively-coupled plasma/mass spectrometry, calibrated with multi-element standards (EM Science, EM Industries Inc., Newark, NJ, USA). Quality assurance and control were conducted with simultaneous analysis of samples of the reference urine Lyphochek^®^ (Bio-Rad, Sydney, Australia), which contained low- and high-range Cd levels. The coefficient of variation of 2.5% was obtained for Cd in the reference urine. Cd concentrations of urine samples reported below the limit of detection (LOD) of 0.05 μg/L were assigned as the LOD divided by the square root of 2. For the Mae Sot group [13], urinary Cd concentrations were determined with an atomic absorption spectrophotometer (Shimadzu Model AA-6300, Kyoto, Japan). Urine standard reference material No. 2670 (The National Institute of Standards, Washington, DC, USA) was used for quality assurance and control purposes.

### 2.3. Clinical Kidney Function Measure and Assessment of Tubular and Glomerular Integrity

Clinical kidney function measure was based on estimated glomerular filtration rate (eGFR), calculated with the Chronic Kidney Disease Epidemiology Collaboration (CKD-EPI) equation [28]. Male eGFR = 141 × [serum creatinine ÷ 0.9]^Y^ × 0.993^age^, where Y = −0.411 if serum creatinine ≤ 0.9 mg/dL, Y= −1.209 if serum creatinine > 0.9 mg/dL. Female eGFR = 144 × [serum creatinine÷0.7]^Y^ × 0.993^age^, where Y= −0.329 if serum creatinine ≤ 0.7 mg/dL, Y= −1.209 if serum creatinine > 0.7 mg/dL. CKD is defined as eGFR < 60 mL/min/1.73 m^2^, and CKD stages I, II, III, IV and V correspond to eGFR 90–119, 60–89, 30–59, 15–29 and <15 mL/min/1.73 m^2^, respectively [28].

Assessment of tubular dysfunction was based on a reduction in tubular reabsorption activity, reflected by an increase in urinary excretion rate of β2-MG [12,13,14,15]. Due to a small molecular weight, β2-MG is filtered, reabsorbed by tubules, and approximately 0.3% of filtered β2-MG is excreted in urine [2]. Assessment of tubular integrity was based on urinary excretion of the enzyme NAG [12,13,14,15] and urinary NAG excretion is considered to be proportional to nephron numbers as this enzyme originates mostly from tubular epithelial cells which is released upon cell injury [2]. For the Bangkok group, the urinary β2-MG assay was based on the latex immunoagglutination method (LX test, Eiken 2MGII; Eiken and Shionogi Co., Tokyo, Japan), and the urinary NAG assay was based on an enzymatic reaction and colorimetry. The urinary protein assay was based on turbidimetry (Roche/Hitachi 717, Boehringer Mannheim and Roche Diagnostics, Roche Diagnostics GmbH Mannheim, Germany). The urinary and serum creatinine assay was based on the Jaffe’s reaction.

For Mae Sot group, the urinary β2-MG assay was based on an enzyme immunoassay (GLAZYME β2 microglobulin-EIA test kit, Sanyo Chemical Industries, Ltd., Kyoto, Japan), while the urinary NAG assayed was based on colorimetry (NAG test kit, Shionogi Pharmaceuticals, Sapporo, Japan). The urinary protein assay was based on the Kingsbury-Clark method, while the urinary and serum creatinine assay was based on the Jaffe’s reaction.

### 2.4. Statistical Analysis

The SPSS statistical package 17.0 (SPSS Inc., Chicago, IL, USA) was used to analyze data. We used the Mann-Whitney U-test to compare two groups of subjects. The distribution of the variables was examined for skewness and those showing right skewing were subjected to logarithmic transformation before analysis, where required. One sample Kolmogorov-Smirnov test was used to detect a departure from normal distribution of variables. We used the logistic regression analysis to estimate Prevalence Odds Ratio (POR) for CKD, attributable to Cd exposure and kidney tubular pathologies. The univariate analysis was used to estimate effect size of Cd exposure levels with adjustment for covariates and urinary Cd quartiles × smoking × gender interactions. In addition, we used a multilinear regression analysis to evaluate the strength of associations between eGFR and its predictors in subjects stratified by gender, smoking status and Cd exposure levels. *p* values ≤ 0.05 for a two-tailed test was considered to indicate statistical significance.

## 3. Results

### 3.1. Characteristics of Study Subjects

Of 395 study subjects, 202 were women and 193 were men. The mean age of women was 4 years older than the mean age of men of 47.4 years (*p* = 0.024). Smoking was more prevalent in men than women (66.8% vs. 24.3%) (*p* < 0.001). The mean (SD) values for eGFR were 86.9 (24.2) mL/min/1.78 m^2^ (range: 19.6–137.8). The CKD prevalence was 13% in men and 12.4% in women (*p* = 0.863), while hypertension prevalence was 24.2% in men and 19.7% in women (*p* = 0.240).

The mean urinary creatinine concentrations in men was higher than women (*p* < 0.001). The mean urinary Cd concentrations in men (7.48 μg/L) and women (5.87 μg/L) did not differ (*p* = 0.930). The mean (SD) urinary Cd was 5.93 (7.69) μg/g creatinine (range: 0.05–57.57). The mean urinary Cd tended to be higher in women than men, when data were adjusted for urine dilution by creatinine excretion (6.41 vs. 5.43 μg/g creatinine, *p* = 0.061). The prevalence of urinary Cd levels above 5.24 μg/g creatinine was 40.3%, while more than half (55.9%) of the subjects had urinary Cd levels, exceeding 1 μg/g creatinine. The prevalence of severe and irreversible tubular dysfunction (urinary β2-MG levels ≥ 1000 μg/g creatinine) was 17.1% in men and 14. 2% in women (*p* = 0.104). Urinary β2-MG, NAG and protein levels in men and women did not differ.

In all subjects, creatinine-adjusted urinary Cd levels showed a strong correlation with age (Spearman rank’s correction coefficient (*r*) = 0.644, *p* = < 0.001), and this association between age and urinary Cd levels persisted after stratification by smoking status (*r* = 0.627, *p* < 0.001 for non-smokers, *r* = 0.540, *p* < 0.001 for smokers). There was an inverse correlation between urinary Cd levels and BMI (*r* = −0.214, *p* < 0.005) in all subjects. After controlling for age, the association of urinary Cd levels and BMI persisted in smokers only (*r* = −0.166, *p* = 0.027), while there was a tendency for an association in non-smokers (*r* = −0.119, *p* = 0.081).

The prevalence rates of various diseases reported by participants differed in men and women (Likelihood Chi-square 15.5, *p* = 0.03). Osteoporosis was more prevalent in women than men (5.6% vs. 0.5%, *p* = 0.004). Kidney disease diagnosis tended to be higher in men than women (4.7% vs. 1.5%, *p* = 0.083).

### 3.2. CKD Prevalence Associated with Tubular Dysfunction and Cadmium Exposure

By logistic regression analysis of CKD prevalence (Table 2), an increase in CKD prevalence odds was found to be associated with age (*p* < 0.001), BMI (*p* = 0.001), tubular dysfunction (urinary β2-MG levels ≥ 300 μg/g creatinine) (*p* = 0.001), urinary Cd (*p* = 0.037) and protein levels (*p* = 0.023). Elevated β2-MG levels associated with the highest increase in CKD prevalence odds (POR 5.324, 95% CI: 2.035, 13.928), followed by urinary Cd levels (POR 2.978, 95% CI: 1.066, 8.317), urinary protein (POR 1.900, 95% CI: 1.093, 3.302), BMI (POR 1.188, 95% CI: 1.071, 1.318), and age (POR 1.119, 95%CI: 1.070, 1.170). Urine NAG did not associate with CKD prevalence (*p* = 0.744).

### 3.3. Effect Size Estimates

Table 3 provides results of a univariate analysis that quantified the variation in eGFR attributable to various independent variables and their interactions. Factors and covariates in the first column accounted for more than a half (67. 3%, *p* < 0.001) of the total eGFR variation. Age accounted for the largest proportion (36.4%) of eGFR variability (*p* < 0.001), while BMI, Cd quartiles, and urine β2-MG each accounted for 2.7% (*p* = 0.001), 5.1% (*p* < 0.001) and 3.3% (*p* < 0.001) in eGFR variation among study subjects. Gender, smoking, urine NAG and protein did not contribute significantly to eGFR variation. There was a significant interaction between gender × smoking that contributed to 2.9% (*p* = 0.011) of eGFR variation. There was a tendency for Cd quartiles × smoking interaction (0.8%, *p* = 0.076).

**Figure 1:**
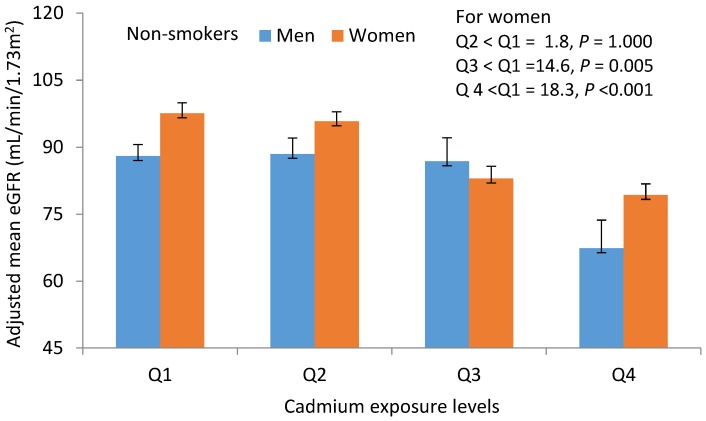
Cadmium-dose dependent reduction in glomerular filtration rates in non-smoking women. Bars represent mean eGFR ± SE values for groups of subjects stratified according to the quartiles of urinary Cd excretion levels. Urinary Cd levels in quartiles 1, 2, 3 and 4 are 0.05–0.50, 0.51–2.95, 2.96–8.80, 8.81–57.57 μg/g creatinine, and the corresponding numbers of subjects are 100, 101, 97 and 97, respectively. The mean eGFR values are adjusted for interactions and covariates as follows; age 48.79 years, BMI 22.21 kg/m^2^, urinary β2-MG 59.74 μg/g creatinine, NAG 4.29 units/g creatinine, and protein excretion 24.98 mg/g creatinine. *p* values ≤ 0.05 indicate statistically significant difference between adjusted mean eGFR in quartile 2, 3 and 4, compared with the urinary Cd quartile 1.

Adjusted mean eGFR across urinary Cd quartiles 1, 2, 3 and 4 were shown separately for male and female non-smokers (Figure 1), given a significant gender × smoking interaction (Table 3). eGFR reduction was associated with urinary Cd levels in a dose-dependent manner in non-smoking women, but not in men. The adjusted mean eGFR [SE] values for urinary Cd quartiles 1, 2, 3 and 4 in non-smoking women were 97.6 [2.3], 95.8 [2.1], 82.9 [2.7], and 79.3 [2.5] mL/min/1.73 m^2^, respectively. The adjusted mean eGFR [SE] in urinary quartile 4, 3 and 2 was 18.3 [3.5] (*p* < 0.001), 14.6 [3.6] (*p* = 0.005) and 1.7 [2.9] (*p* = 1.000) mL/min/1.73 m^2^ lower than the adjusted mean eGFR in urinary Cd quartile 1, respectively. The total number of non-smoking women was 153 and the numbers (%) distribution in urinary Cd quartiles 1, 2, 3 and 4 were 42 (27.5%), 49 (32%), 27 (17.6%), 35 (22.9%), respectively. The total number of non-smoking men was 64 and the number (%) distribution in urinary Cd quartile 1, 2, 3 and 4 were 36 (56.3%), 16 (25%), 7 (10.9%), and 5 (7.8%), respectively. In non-smoker male group, adjusted mean eGFR, the adjusted mean eGFR [SE] in urinary quartile 4, 3 and 2 was 20.7 [6.8] (*p* = 0.193), 21.1 [7.1] (*p* = 0.251) and 19.5 [7.6] (*p* = 1.000) mL/min/1.73 m^2^ lower than the adjusted mean eGFR in urinary Cd quartile 1, respectively.

### 3.4. Evidence for Urinary Cd Threshold Level

Table 4 shows results of a multilinear regression, used to further explore associations of eGFR, and kidney pathology markers. Age, BMI, gender, smoking, urinary Cd, β2-MG, NAG, and protein levels accounted for 66.5% of the total eGFR variation among study subjects. Age showed the strongest inverse association with eGFR (β = −0.548, *p* < 0.001), followed by urine Cd (β = −0.234, *p* < 0.001), β2-MG (β = −0.178, *p* < 0.001) and BMI (β = −0.105, *p* = 0.001). There was a marginal association between eGFR and female gender (β = 0.066, *p* = 0.051). Associations of eGFR and urinary NAG (β = 0.004, *p* = 0.893) and protein levels (β = −0.037, *p* = 0.236) were not significant.

**Figure 2:**
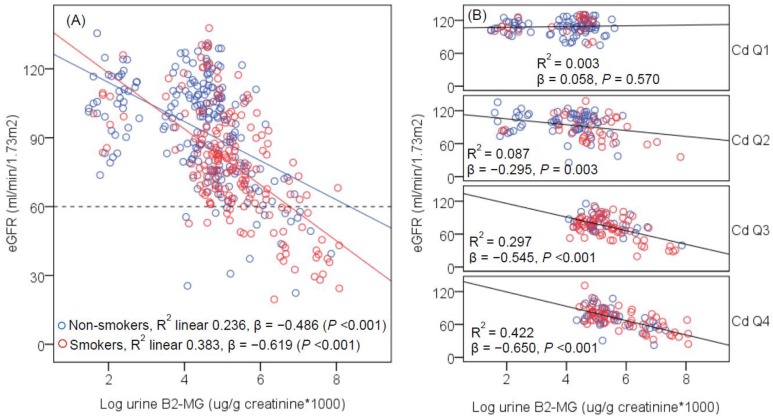
Scatterplots of kidney function measure vs. tubular dysfunction biomarker. The regression lines of eGFR vs. urinary β2-MG levels are shown for groups of subjects according to smoking status (**A**) and urinary Cd quartiles (**B**). The reference line in (**A**) is based on the CKD diagnosis, eGFR < 60 mL/min/1.73 m^2^. The *R*^2^ values and the β coefficients shown in (**A**,**B**) are unadjusted. Urinary Cd levels in quartiles 1, 2, 3 and 4 are 0.05–0.50, 0.51–2.95, 2.96–8.80, and 8.81–57.57 μg/g creatinine, and the corresponding numbers of subjects are 100, 101, 97 and 97, respectively.

**Figure 3:**
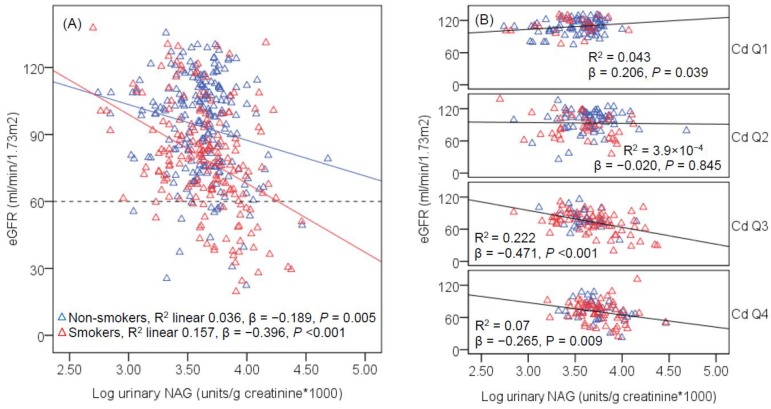
Scatterplots of kidney function measure vs. tubular injury biomarker. The regression lines of eGFR vs. urinary NAG levels are shown for groups of subjects according to smoking status (**A**) and urinary Cd quartiles (**B**). The reference line in (**A**) is based on the CKD diagnosis, eGFR < 60 mL/min/1.73 m^2^. The *R*^2^ values the β coefficients shown in (**A**,**B**) are unadjusted. Urinary Cd levels in quartiles 1, 2, 3 and 4 are 0.05–0.50, 0.51–2.95, 2.96–8.80, and 8.81–57.57 μg/g creatinine, and the corresponding numbers of subjects are 100, 101, 97 and 97, respectively.

**Figure 4:**
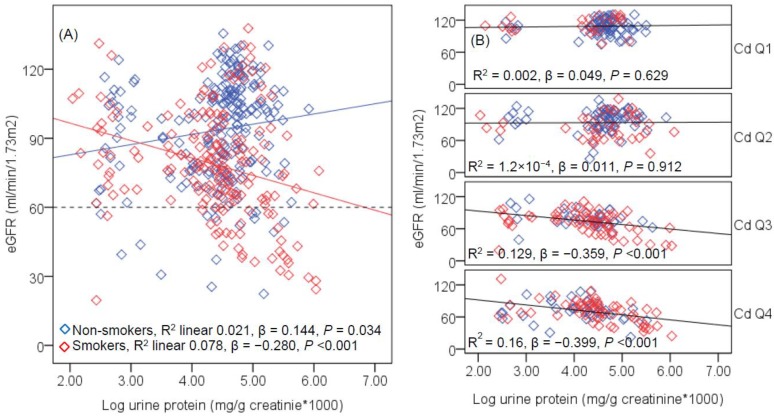
Scatterplots of kidney function measure vs. glomerular damage biomarker. The regression lines of eGFR vs. urinary protein levels are shown for subjects stratified according to smoking status (**A**) and urinary Cd quartiles (**B**). The reference line in (**A**) is based on the CKD diagnosis, eGFR < 60 mL/min/1.73 m^2^. The *R*^2^ values and the β coefficients shown in (**A**,**B**) are unadjusted. Urinary Cd levels in quartiles 1, 2, 3 and 4 are 0.05–0.50, 0.51–2.95, 2.96–8.80, and 8.81–57.57 μg/g creatinine, and the corresponding numbers of subjects are 100, 101, 97 and 97, respectively.

Figure 2, Figure 3 and Figure 4 provide data for the strength (β) of associations of eGFR and kidney pathology markers (urine β2-MG, NAG, and protein) across urinary Cd quartiles. In all subjects (Figure 2), a strongly inverse association was seen between eGFR and β2-MG in both non-smokers (β = −0.486, *p* < 0.001) and smokers (β = −0.619, *p* < 0.001). In unadjusted models, eGFR was not associated with β2-MG in Cd exposure quartile 1 (β = 0.058, *p* = 0.570), but in quartile 2 (β = −0.295, *p* = 0.003), quartile 3 (β = −0.545, *p* < 0.001) and quartile 4 (β = −0.650, *p* < 0.001). After adjustment for covariates and interactions; the β coefficients (*p* values) of the eGFR and β2-MG association in Cd exposure quartiles 1, 2, 3 and 4 were 0.013 (*p* = 0.897), −0.246 (*p* = 0.020), −0.547 (*p* < 0.001), and −0.685 (*p* < 0.001), respectively.

In all subjects (Figure 3), a marginally inverse association between eGFR and NAG was seen in non-smokers (β = −0.189, *p* = 0.005), while a moderately inverse association was in smokers (β = −0.396, *p* < 0.001). In unadjusted models, a marginally positive association between eGFR and NAG was evident in urinary Cd quartile 1 (β = 0.206, *p* = 0.039), eGFR and NAG association was absent in quartile 2 (β = −0.020, *p* = 0.845). A strongly inverse association was seen between eGFR and NAG in quartile 3 (β = −0.471, *p* < 0.001), while a moderately inverse association existed in quartile 4 (β = −0.265, *p* = 0.009). After adjustment for covariates and interactions, the β coefficient (*p* value) of eGFR and NAG association in Cd exposure quartiles 1, 2, 3 and 4 were 0.190 (*p* = 0.057), −0.061 (*p* = 0.546), −0.445 (*p* < 0.001), and −0.271, (*p* = 0.009), respectively.

In all subjects (Figure 4), a marginally positive association was seen between eGFR and urinary protein in non-smokers (β = 0.144, *p* = 0.034), but a moderately inverse association was seen in smokers (β = −0.280, *p* < 0.001). In unadjusted models, an association of eGFR and urine protein was not present in urinary Cd quartile 1 (*p* = 0.629) and quartile 2 (*p* = 0.912), while a moderately inverse association was evident in quartile 3 (β = −0.359, *p* < 0.001) and quartile 4 (β = −0.399, *p* < 0.001), After adjustment for covariates and interactions, the β coefficients (*p* values) of eGFR and protein associations in urinary Cd quartiles 1, 2, 3 and 4 were 0.062 (*p* = 0.565), 0.013 (*p* = 0.894), −0.350 (*p* < 0.001), and −0.413 (*p* < 0.001), respectively.

## 4. Discussion

Herein, we have observed for the first time an association of a 5.32-fold rise in CKD prevalence odds and urinary β2-MG levels ≥ 300 μg/g creatinine in Thai subjects with chronic environmental exposure to Cd. This independent association between elevated levels of urinary β2-MG and a marked increase in odds of CKD prevalence suggests a vital role played by kidney tubular cells in the pathogenesis and/or progression of CKD. Indeed, a tubular-glomerular connection is increasingly recognized [31] as is the evidence for β2-MG as marker of a range of kidney disease [32,33,34]. Our finding concurs with experimental data and clinical outcomes that suggest urinary β2-MG is a predictor of GFR reduction [32,33,34].

Supporting tubular-glomerular connection are data from a prospective cohort study in Japan showing that a sign of tubular impairment (urine β2-MG levels ≥ 300 μg/g creatinine) was associated with a 79% (95% CI: 1.07, 2.99) increase in the likelihood of having eGFR fall at high rates, i.e., 10 mL/min/1.73 m^2^ over 5-year observation period [35]. In another cross-sectional study, a milder tubular impairment (urine β2-MG levels ≥ 145 μg/g creatinine) was associated with an increase in the prevalence odds for hypertension in Japanese subjects [36]. Results of these Japanese studies underscored clinical values of urine β2-MG measurement, but Cd exposure levels experienced by Japanese subjects in these two studies were not measured. Thus, it is unknown if these observed outcomes (rapid GFR reduction and hypertension development) in subjects with high urine β2-MG levels could be linked to Cd or other environmental factors.

Urinary Cd levels > 1 μg/L (>0.5 μg/g creatinine) were associated with a 48% increase in the risk of CKD development (95% CI: 1.01, 2.17) in adult participants in the U.S. NHANES 1999–2006 cycle [21]. Consistent with the U.S. study is our finding of an association between elevated Cd body burden, assessed by urinary Cd levels, and an increase in odds of CKD prevalence (2.98 fold). Multilinear regression data indicated also that lower eGFR values were associated with higher urinary Cd levels. Further, in an effect-size analysis, a dose-response between eGFR reduction and urinary Cd quartiles was evident in non-smoking women. This may implicate dietary Cd intake in the pathogenesis of CKD. Likewise, in a Chinese population study, cumulative Cd intake estimate was associated with a 4-fold increase in CKD prevalence (95% CI: 2.91, 5.63) [25].

An association of lower eGFR and higher blood Cd levels was noted in Korean population [37] and the representative of the U.S. population (the U.S. NHANES, 2007–2012) [38]. In a Korean study, blood Cd levels in the highest tertile were associated with 1.85 mL/min/1.73 m^2^ lower GFR values (95% CI: −3.55, −0.16), compared with the lowest tertile [37]. However, it is noteworthy the majority of Cd in blood is in red blood cells, which are not filtered (not present in glomerular filtrate) [2]. Consequently, it is impossible to attribute blood Cd to eGFR reduction and to Cd toxicity in the kidney in the absence of data on kidney pathology. A 2.91-fold increase in CKD risk (95% CI: 1.76, 4.81) was associated with blood Cd levels > 0.6 μg/L in the U.S. NHANES 1999–2006 adult participants [23]. Blood Cd levels > 0.53 μg/L were associated with an approximately two-fold increase in risk of CKD development (95% CI: 1.09, 4.50) among adult participants in the NHANES 2011–2012 [22]. In the Korean population study, elevated blood Cd levels, but not blood Pb or blood Hg, were associated with CKD, especially in those with hypertension [24].

Currently, urinary Cd threshold limit for CKD is lacking. However, there are several urinary Cd threshold limits that have been derived for kidney tubular toxicity using benchmark dose method [2,39]. In one study, urinary Cd 0.57–1.84 μg/g creatinine was identified as threshold levels for urinary β2-MG levels ≥ 1065 μg/g creatinine [14]. As discussed above, it was evident that GFR and CKD both were substantially associated with elevated urine β2-MG and that association of GFR and β2-MG was minimal (or absent) in subjects with urinary Cd in quartile 1 (urinary Cd 0.05–0.50 μg/g creatinine). In the absence of threshold limit for CKD and a continuously rising CKD prevalence worldwide, it is argued that urinary Cd of 0.50 μg/g creatinine might be useful. Urinary Cd of 0.50 μg/g creatinine is 2-fold and 10-fold lower than the threshold level for kidney Cd toxicity, established by the European Food Safety Agency [29] and the WHO/FAO [30], respectively. Urinary Cd levels < 1 μg/g creatinine have been found to be associated with kidney pathologies in many previous studies [3,4]. In a study of Swedish women, 53–64 years of age, urinary Cd of 0.67 μg/g and 0.8 μg/g creatinine were found to be associated with markers of tubular impairment and glomerular dysfunction, respectively [40]. Urinary Cd of 0.74 μg/g creatinine was associated with albuminuria in the Torres Strait (Australia) women who had diabetes [41].

## 5. Conclusions

For the first time, we have demonstrated that a clinical kidney function measure such as estimated glomerular filtration rates could be linked to both Cd exposure and tubular toxicity in Cd-dose and toxicity severity dependent manner. In addition, we have shown that a urinary Cd level as low as 0.50 μg/g creatinine might be used as a warning sign of excessive Cd intake, Cd toxic burden, kidney pathologies and kidney function deterioration. Urinary Cd of 0.50 μg/g creatinine is 10-fold lower than current threshold for kidney toxicity established by the FAO/WHO of 5.24 μg/g creatinine. This established urinary Cd threshold level does not afford health protection. Consequently, there is an urgent need to reassess Cd toxic burden and urinary Cd toxicity threshold limit that should prevent human population from excessive Cd exposure, and CKD development.

## 6. Strengths and Limitations

The strengths of this study include the samples of men and women with homogeneous exposure sources (i.e., none were occupationally exposed) together with a wide Cd-exposure range (urinary Cd 0.05–58 μg/g creatinine) and a wide eGFR range (19.6–137.8 mL/min/1.73 m^2^) suitable for dose-response relationship analysis. The high CKD prevalence of 12.7% in villages with varying degrees contamination allowed recruitment of sufficient numbers of subjects with low GFR and CKD. The community-based recruitment strategy minimized bias toward certain subpopulation groups, frequently encountered in health center-based studies.

The limitations of this study were its small sample size and its cross-sectional design, which limited an assessment of temporal relationships between variables or causal inference of Cd exposure. A wide age range was another limitation as GFR falls with increasing age due to loss of nephrons [2]. GFR could also fall due to tubular pathologies induced by Cd and other environmental nephrotoxicants. Most subjects with high-Cd exposure were rice farmers, co-exposure to other nephrotoxicants in pesticides might also be a possible confounder. Heavy smoking, and presence of disease notably hypertension and diabetes were likely confounders. GFR may fall because of kidney damage due to smoking. This was evident in Figure 2, where an additional effect of smoking on eGFR was suggested by the increasing β slope in urinary Cd quartiles 3 and 4, relative to quartiles 1 and 2, given the higher prevalence of smokers in urinary quartile 3 (35.4%) and quartile 4 (32%), compared to quartile 2 (20.2%) and quartile 1 (12.4%).

GFR may also fall because of kidney damage due to hypertension, and because of nephron loss, urinary excretion of NAG in heavy smokers, hypertensive and diabetic subjects could be lower than expected. This was evident in Figure 3, where there was a marked drop in the β slope in quartile 4, compared with quartile 3. Such a drop in β slope could be interpreted to be resulted from loss of tubular cells, leading to lower urinary NAG excretion levels than expected in quartile 4.

Urinary Cd concentrations were determined by two methods. For low-Cd exposure group, a high sensitive and high specificity method, known as inductively-coupled plasma mass spectrometry, was used. A less sensitive, but sufficiently high specificity assay with atomic absorption spectrophotometer was used for high-Cd exposure group. However, data from quality control and assurance conduced with standard urine specimens suggest that variation due to different methods was relatively small. 

## Figures and Tables

**Table 1:** Characteristic of study subjects.

**Descriptors/Variables**	**All Subjects** ***n* = 395**	**Men** ***n* = 193**	**Women** ***n* = 202**	***p* Values**
Age (years)	48.8 ± 14.0	47.4 ± 15.8	50.1 ± 12.0	0.024
BMI (kg/m^2^)	22.2 ± 3.8	22.0 ± 3.4	22.4 ± 4.1	0.387
Smoking prevalence (%) a	45.1	66.8	24.3	<0.001
Hypertension (%) ^b^	21.7	24.2	19.3	0.240
eGFR (mL/min/1.73 m^2^)	86.9 ± 24.2	87.2 ± 25.0	86.6 ± 23.5	0.717
CKD prevalence (%)	12.7	13	12.4	0.863
**Exposure indicators**				
Urinary creatinine (mg/dL)	100.2 ± 67.7	115.1 ± 71.7	85.8 ± 60.5	<0.001
Urinary Cd concentration (μg/L)	6.65 ± 10.70	7.48 ± 12.71	5.87 ± 8.29	0.930
Urinary Cd (μg/g creatinine)	5.93 ± 7.69	5.43 ± 7.60	6.41 ± 7.77	0.061
Urinary Cd >1 μg/g creatinine (%) ^c^	55.9	53.4	58.4	0.312
Urinary Cd >5.24 μg/g creatinine (%) ^d^	40.3	37.3	43.1	0.243
**Renal pathology markers**				
β2-MG (mg/g creatinine)	2.68 ± 12.43	3.35 ± 13.87	2.04 ± 10.88	0.973
β2-MG ≥ 1 mg/g creatinine (%) ^e^	14.2	17.1	11.4	0.104
NAG (Units/ g creatinine)	5.31 ± 4.26	4.98 ± 3.50	5.63 ± 4.86	0.103
Total protein (mg/g creatinine)	75.6 ± 142	74.7 ± 144	76.4 ± 141	0.200
**Reported health status (%)**				
No disease	66.7	67.4	66.0	0.901
Anemia	6.2	4.7	7.6	0.221
Hypertension	15.5	16.3	14.7	0.796
Diabetes	2.8	3.2	2.5	0.763
Osteoporosis	3.1	0.5	5.6	0.004
Kidney disease	3.1	4.7	1.5	0.083
Urinary stones	1.6	1.6	1.5	1.000
Others	1.0	1.6	0.5	0.317

Numbers are arithmetic mean ± standard deviation (SD). eGFR is determined with CKD−EPI equation, and eGFR < 60 mL/min/1.73 m^2^ is defined as CKD [28]. ^a^ Both current and ex-smokers are grouped together because of a known long half-life of Cd in the body. ^b^ Hypertension was defined as systolic blood pressure ≥ 140 mmHg, or diastolic blood pressure ≥ 90 mmHg, physician diagnosis, or prescription of anti-hypertensive medications. ^c^ Tubular Cd toxicity threshold, established by the European Food Safety Agency [29]. ^d^ Tubular Cd toxicity threshold, established by the FAO/WHO [30]. ^e^ Severe and irreversible tubular dysfunction [19,20].

**Table 2:** Increased prevalence odds of chronic kidney disease associated with cadmium and severity of tubular dysfunction.

Independent Variables	POR of CKD	95% CI for POR		*p* Values
		Lower	Upper	
Gender	0.771	0.317	1.876	0.566
Age (years)	1.119	1.070	1.170	<0.001
BMI (kg/m^2^)	1.188	1.071	1.318	0.001
Smoking	1.002	0.378	2.661	0.996
Tubular dysfunction ^a^	5.324	2.035	13.928	0.001
Log urine Cd (μg/g creatinine)	2.978	1.066	8.317	0.037
Log urine NAG (units/g creatinine)	1.340	0.231	7.770	0.744
Log urine protein (mg/g creatinine)	1.900	1.093	3.302	0.023

POR = Prevalence Odds Ratio. ^a^ Tubular dysfunction is defined as urinary β2-MG levels ≥ 300 μg/g creatinine [19,20]. POR was derived from a logistic regression model analysis in which CKD (eGFR < 60 mL/min/1.73 m^2^) was a categorical dependent variable, while age, BMI, creatinine adjusted urinary Cd, NAG and protein levels were continuous independent variables. Categorical independent variables were gender, smoking status, and tubular dysfunction. *p* values ≤ 0.05 are considered to indicate statistical significant levels.

**Table 3:** Univariate analysis of glomerular filtration rates.

Factors and Covariates	Degree of Freedom	eGFR (mL/min/1.73 m^2^)		
		*F*	*p*	η^2^
Corrected Model	19	43.715	<0.001	0.689
Intercept	1	219.823	<0.001	0.370
Age (years)	1	214.578	<0.001	0.364
BMI (kg/m^2^)	1	10.484	0.001	0.027
Smoking	1	0.082	0.775	0.000
Gender	1	1.347	0.247	0.004
Log urine β2-MG (μg/g creatinine)	1	12.800	<0.001	0.033
Log urine NAG (units/g creatinine)	1	0.275	0.600	0.001
Log urine protein (mg/g creatinine)	1	2.405	0.122	0.006
Urinary Cd quartiles	3	6.765	<0.001	0.051
Gender × smoking	1	3.161	0.076	0.008
Urinary Cd quartiles × smoking	3	3.747	0.011	0.029
Urinary Cd quartiles × gender	3	0.890	0.446	0.007
Urinary Cd quartiles × gender × smoking	2	2.032	0.133	0.011
Error	375			
Total	395			
Corrected Total	394			

Adjusted *R*^2^ = 0.673; η^2^ = eta squared. Adjusted *R*^2^ value describes the total eGFR variability attributable to all factors and covariates. The η^2^ value describes the proportion of eGFR variability attributable to each factor/covariate. eGFR in mL/min/1.73 m^2^ was a continuous dependent variable. Age, BMI, creatinine-adjusted urinary β2-MG, NAG, and protein excretion levels were continuous independent variables. Gender, smoking status and Cd exposure levels were categorical independent variables. *p* values ≤ 0.05 are considered to indicate statistical significant levels. Quartiles 1, 2, 3, and 4 of Cd exposure levels correspond to urinary Cd; 0.05–0.50, 0.51–2.95, 2.96–8.80, 8.81–57.57 μg/g creatinine, respectively. The numbers of subjects in quartile 1, 2, 3 and 4 were 100, 101, 97, and 97, respectively.

**Table 4:** Multilinear regression analysis of glomerular filtration rates.

Independent Variables	eGFR (mL/min/1.73 m^2^)			
	Standardized	95% CI for β		*p* Value
	β coefficients	Lower	Upper	
Age (years)	−0.548	−1.084	−0.812	<0.001
BMI (kg/m^2^)	−0.105	−1.068	−0.288	0.001
Gender	0.066	−0.017	6.435	0.051
Smoking	−0.002	−3.543	3.397	0.967
Log urine Cd (μg/g creatinine)	−0.234	−10.900	−4.976	<0.001
Log urine β2-MG (μg/g creatinine)	−0.178	−4.773	−1.808	<0.001
Log urine NAG (units/g creatinine)	0.004	−5.042	5.780	0.893
Log urine protein (mg/g creatinine)	−0.037	−2.982	0.736	0.236

Adjusted *R*^2^ = 0.665, *p* < 0.001. eGFR was a continuous dependent variable. Gender (male = 1, female = 2), smoking (non-smoker = 1, smoker = 2) were categorical independent variables, while age, BMI, creatinine adjusted urinary Cd, NAG and protein levels were continuous independent variables. *p* values ≤ 0.05 are considered to indicate statistical significant levels.
